# A Bifunctional Adsorber Particle for the Removal of Hydrophobic Uremic Toxins from Whole Blood of Renal Failure Patients

**DOI:** 10.3390/toxins11070389

**Published:** 2019-07-03

**Authors:** Marieke Sternkopf, Sven Thoröe-Boveleth, Tobias Beck, Kirsten Oleschko, Ansgar Erlenkötter, Ulrich Tschulena, Sonja Steppan, Thimoteus Speer, Claudia Goettsch, Vera Jankowski, Joachim Jankowski, Heidi Noels, The European Uremic Toxin Work Group-EUTox

**Affiliations:** 1 Institute for Molecular Cardiovascular Research (IMCAR), University Hospital Aachen, RWTH Aachen University, 52074 Aachen, Germany; 2 Institute for Occupational, Social and Environmental Medicine; University Hospital Aachen, RWTH Aachen University, 52074 Aachen, Germany; 3 Institute of Inorganic Chemistry, RWTH Aachen University, 52056 Aachen, Germany; 4 PSS Polymer Standards Service GmbH, 55120 Mainz, Germany; 5 Fresenius Medical Care Deutschland GmbH, 66606 St. Wendel, Germany; 6 Fresenius Medical Care Deutschland GmbH, 61352 Bad Homburg, Germany; 7 Department of Internal Medicine—Nephrology and Hypertension, Saarland University Medical Centre, 66421 Homburg/Saar, Germany; 8 Medical Clinic I—Cardiology, University Hospital RWTH Aachen, 52074 Aachen, Germany; 9 School for Cardiovascular Diseases, Maastricht University, 6200 Maastricht, The Netherlands; 10 European Uremic Toxin Work Group-EUTox, Danube University Krems, 3500 Krems, Austria

**Keywords:** activated charcoal, hemodialysis, adsorption, uremic toxins, hemocompatibility, chronic kidney disease

## Abstract

Hydrophobic uremic toxins accumulate in patients with chronic kidney disease, contributing to a highly increased cardiovascular risk. The clearance of these uremic toxins using current hemodialysis techniques is limited due to their hydrophobicity and their high binding affinity to plasma proteins. Adsorber techniques may be an appropriate alternative to increase hydrophobic uremic toxin removal. We developed an extracorporeal, whole-blood bifunctional adsorber particle consisting of a porous, activated charcoal core with a hydrophilic polyvinylpyrrolidone surface coating. The adsorption capacity was quantified using analytical chromatography after perfusion of the particles with an albumin solution or blood, each containing mixtures of hydrophobic uremic toxins. A time-dependent increase in hydrophobic uremic toxin adsorption was depicted and all toxins showed a high binding affinity to the adsorber particles. Further, the particle showed a sufficient hemocompatibility without significant effects on complement component 5a, thrombin-antithrombin III complex, or thrombocyte concentration in blood in vitro, although leukocyte counts were slightly reduced. In conclusion, the bifunctional adsorber particle with cross-linked polyvinylpyrrolidone coating showed a high adsorption capacity without adverse effects on hemocompatibility in vitro. Thus, it may be an interesting candidate for further in vivo studies with the aim to increase the efficiency of conventional dialysis techniques.

## 1. Introduction

A vast amount of substances with disparate physico-chemical properties are accumulating in the blood of patients suffering from chronic kidney disease (CKD), leading to uremic toxicity [1]. The conventional dialysis therapies aim to replace the filtration capacity of the failing kidney by clearing a range of uremic toxins from the blood of CKD patients. However, efficient removal cannot currently be reached for the whole spectrum of uremic toxins, especially for the group of hydrophobic, protein-bound substances. In general, uremic toxins are grouped into three classes based on their molecular weight and chemical properties: (i) small, water-soluble solutes that are not protein-bound (<500 Da), which can be easily removed by dialysis; (ii) middle molecules (>500 Da), which can be cleared using dialysis membranes with larger pore size or hemodiafiltration; and (iii) proteins as well as hydrophobic, protein-bound uremic toxins [1,2,3]. The latter uremic toxins have a molecular weight smaller than 500 Da but are very difficult to remove using conventional dialysis due to their hydrophobicity. The solubility of hydrophobic substances in an aqueous solution is strongly limited (*similia similibus solvuntur*) and consequently, the substances accumulate in the plasma of patients. In addition, their high binding affinity towards plasma proteins, such as albumin, substantially increases their molecular weight and prevents their efficient removal through the pores of the dialysis membranes [2,3]. For example, studies have shown that ≈70% of phenylacetic acid [4], ≈90% of p-cresyl sulfate [5], and >90% of indoxyl sulfate [6] are protein-bound in the blood. In healthy subjects, the tubular epithelial cells within the kidney facilitate the removal of these hydrophobic uremic toxins from the blood by shifting their bound state to free fraction via various transporters [7]. In contrast, only ≈25% of the protein-bound uremic toxins are removed by dialysis [8]. In recent years, different studies have tested new approaches to improve the removal of protein-bound uremic toxins. However, studies on longer dialysis sessions and hemodiafiltration were only able to achieve moderate improvements [9,10]. Recent studies indicate that these hydrophobic, protein-bound uremic toxins may play an essential role in the high morbidity and mortality of CKD patients, including their highly increased cardiovascular risk, as is clearly shown for p-cresyl sulfate and indoxyl sulfate [11,12,13,14,15]. 

Restoring the blood filtrating function of the kidney is therefore a major challenge and many renal replacement therapies targeting the removal of protein-bound uremic toxins have emerged over the last few decades. Nonetheless, to date, none of these attempts have managed to adequately filtrate protein-bound uremic toxins [9,10,16,17,18], resulting in an ongoing demand for new strategies to efficiently remove protein-bound uremic toxins from blood.

One long-standing idea to improve the conventional high-flux hemodialysis therapy is the use of extracorporeal adsorber particles to mimic plasma proteins in binding hydrophobic uremic toxins. Early studies indicated that hemoperfusion using activated charcoal enabled the removal of uric acid [19], for example, but was clinically not feasible due to a plethora of adverse effects such as extensive deposits of fine charcoal particles, depletion of platelets and leukocytes, and allergic reaction [20]. So far, commercial adsorbers have been analyzed for the treatment of patients with acute intoxication, sepsis, liver failure, and hypercholesterinemia. Also, they have been tested in pilot studies for extracorporeal removal of soluble fms-like tyrosine kinase 1 in preeclampsia [21,22,23]. Further, it was demonstrated that utilizing an extracorporeal liver support adsorption-dialysis device (fractionated plasma separation, adsorption, and dialysis (FPAD) system) was more effective in the removal of protein-bound, hydrophobic uremic toxins than conventional high-flux hemodialysis [16]. However, due to the equipment necessary for FPAD and the associated costs, this approach is not suitable for the regular extracorporeal therapy of CKD patients. A special adsorber for the regular therapy of CKD patients is not yet available as the combination of an efficient adsorption capacity with an adequate hemocompatibility still remains a challenge.

Therefore, in this study, we developed and examined the performance of an activated spherical charcoal particle coated with a hydrophilic, cross-linked polymer for uremic toxin adsorption. This innovative adsorber particle was designed to combine (i) size exclusion, to enable the exclusion of larger plasma proteins; (ii) hydrophobicity, to enable effective binding of hydrophobic uremic toxins; and (iii) hemocompatibility due to the coating with a hydrophilic, cross-linked polymer to achieve usability for whole blood applications. The resulting adsorber particle may be used in both renal or hepatic replacement therapies for detoxification and in the treatment of sepsis in future clinical applications, and might prevent cardiovascular disease in CKD patients.

## 2. Results

### 2.1. Processing and Characterization of the Newly Developed Adsorber Particle

**Figure 1:**
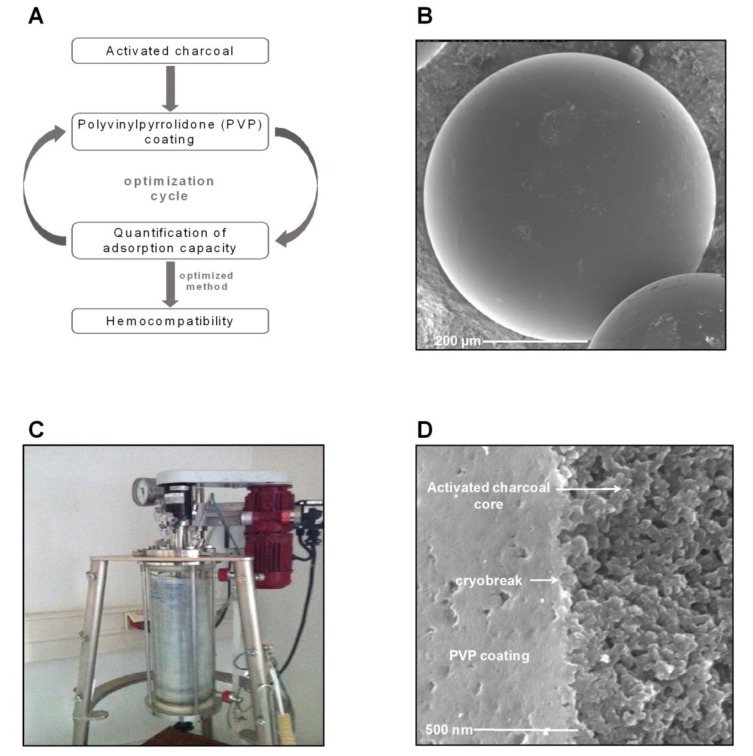
Synthesis of the whole-blood adsorber particle. (**A**) Flowchart of the experimental design for synthesis of the adsorber particles. (**B**) Representative scanning electron microscopic image of the uncoated activated charcoal. (**C**) Representative image of the experimental setup for the suspension polymerization process for coating the activated charcoal core particles. (**D**) Representative scanning electron microscopic image of the newly developed adsorber particle after coating of the charcoal core particles. Scale bars represent 200 µm (**B**) and 500 nm (**D**).

**Figure 2:**
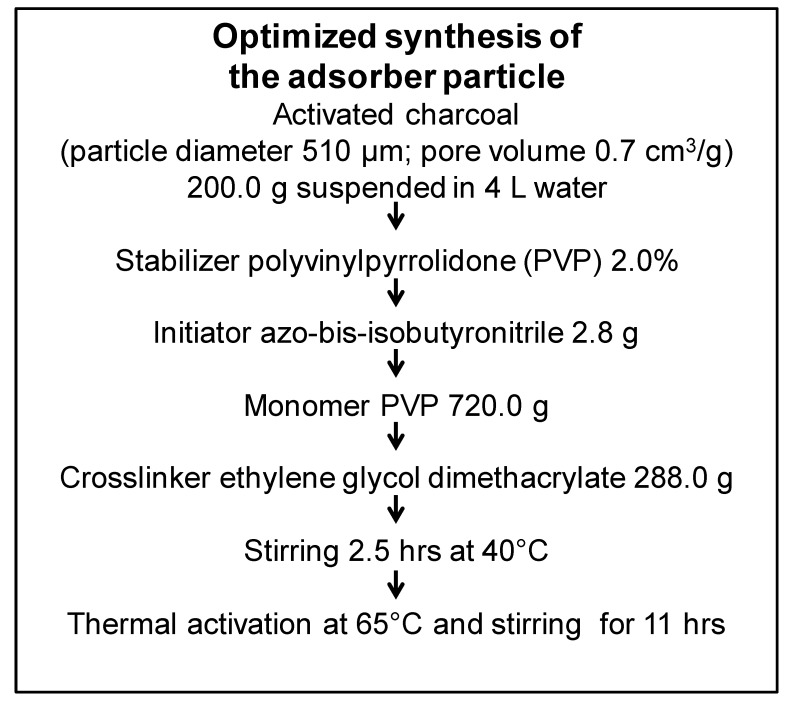
Overview of components and conditions used for the generation of the optimized whole-blood adsorber particle. The particle was generated by coating activated charcoal with polyvinylpyrrolidone (PVP) in a suspension polymerization process using the indicated amounts of stabilizer, initiator, monomer, and crosslinker in the indicated conditions of heating and incubation.

The core of the newly developed adsorber particles consists of activated charcoal, a material that was shown to highly adsorb arsenic, selenium and mercury in aqueous solutions [24]. Polyvinylpyrrolidone (PVP) was previously used as a surface coating for silver nanoparticles due to a good hemocompatibility [25]. The charcoal adsorbent beads were dissolved in a mixture of PVP, crosslinker (ethylene glycol dimethacrylate), and initiator (azo-bis-isobutyronitrile) in water, finally leading to the formation of coated particles through a suspension polymerization process [26]. Systematically, different ratios of monomer and crosslinker were tested, resulting in 13 different particle types. These particles were tested regarding their adsorption capacity toward hydrophobic uremic toxins. Hemocompatibility was analyzed for the particles with a high adsorption capacity (Figure 1A). Finally, one particle was selected for in-depth characterization. Starting from the uncoated charcoal adsorbent particles (Figure 1B), coating with PVP in a 5 L glass reactor through a suspension polymerization process (Figure 1C) resulted in the formation of the selected particle with a highly porous core and a homogenous PVP coating without any surface irregularities (Figure 1D). The optimized and detailed synthesis of this particle is illustrated in Figure 2.

### 2.2. Assessment of the Adsorption Capacity of the Particle towards Hydrophobic, Protein-Bound Uremic Toxins

**Figure 3:**
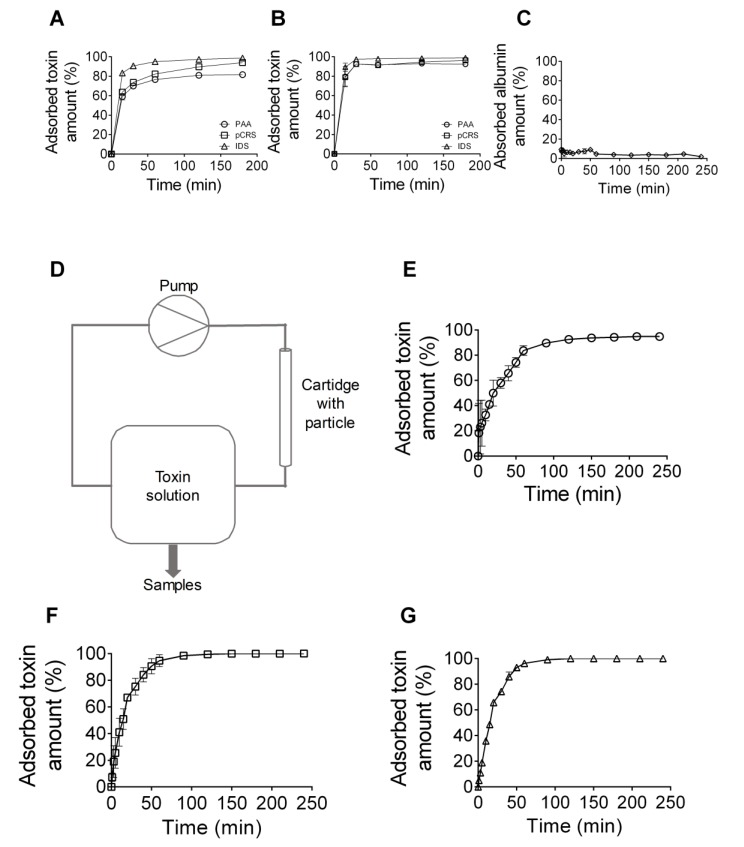
Quantification of the binding capacity of the newly developed adsorber particle to hydrophobic, protein-bound uremic toxins. The particles were incubated with the uremic toxins phenylacetic acid (PAA), p-cresyl sulfate (pCRS), and indoxyl sulfate (IDS), and their adsorption capacities were quantified by reversed-phase chromatography. (**A**,**B**) Quantification of the adsorption capacity after static incubation of the adsorber particles with the uremic toxins dissolved in BSA solution (**A**) or in blood (**B**) for different incubation periods, as indicated. Shown are mean values ± S.E.M of three independent experiments. (**C**) Quantification of the protein concentration of a BSA solution after static incubation with the adsorber particles. Shown are mean values ± S.E.M of four independent experiments. (**D**) Flowchart of the experimental set-up for the quantification of uremic toxin adsorption to adsorber particles in conditions of flow. (**E**–**G**) Quantification of the adsorption capacity after flow incubation of the adsorber particles with phenylacetic acid (PAA) (**E**), p-cresyl sulfate (pCRS) (**F**), or indoxyl sulfate (IDS) (**G**), all dissolved in PBS, and after different time points, as indicated. Shown are mean values ± S.E.M of three independent experiments.

The capacity of the generated particles to adsorb hydrophobic uremic toxins was analyzed after time-dependent incubation of the particles with selected hydrophobic uremic toxins using analytical reversed phase high performance liquid chromatography (RP-HPLC). The experiments depicted a time-dependent increase in the adsorbed toxin amount for all uremic toxins investigated (phenylacetic acid, p-cresyl sulfate, and indoxyl sulfate), either when solved in BSA solution (Figure 3A) or blood (Figure 3B). Each of the three uremic toxins dissolved in a BSA solution was very efficiently adsorbed to the particle, demonstrating a mean adsorption level of 91.4 ± 5.1% after 180 min of incubation (phenylacetic acid 81.7 ± 1.2%, p-cresyl sulfate 94.0 ± 0.5%, indoxyl sulfate 98.7 ± 0.3%) (Figure 3A). When present in blood, all three uremic toxins showed a high affinity to the particle, with in average 94.2 ± 1.7% of the toxins adsorbed even after only 30 min of incubation (phenylacetic acid 92.5 ± 1.4%, p-cresyl sulfate 92.5 ± 1.4%, indoxyl sulfate 97.5 ± 0.9%) (Figure 3B). In contrast, the particles did not show a significant adsorption of BSA even up to an incubation time of 240 min (Figure 3C).

Furthermore, the adsorption capacity of the absorber particles towards hydrophobic uremic toxins was quantified regarding the conditions of flow. The absorber particles were packed into a cartridge and 1 L of aqueous PBS containing a mixture of the uremic toxins phenylacetic acid, p-cresyl sulfate, and indoxyl sulfate was pumped through the cartridge (Figure 3D). Analysis of the samples, collected at the outlet of the cartridge at different time points, demonstrated that after 60 min, the uremic toxins phenylacetic acid, p-cresyl sulfate, and indoxyl sulfate were adsorbed 83.6 ± 2.2%, 94.7 ± 2.6%, and 96.3 ± 1.8%, respectively. After 240 min, the removal efficiencies reached 94.8 ± 1.4% (for phenylacetic acid) (Figure 3E) and 100.0 ± 0.0% (for p-cresyl sulfate and indoxyl sulfate) (Figure 3F,G). 

In summary, the newly developed absorber particle is highly efficient at adsorbing hydrophobic, protein-bound uremic toxins that are present in the blood of patients with CKD stage 5, both under static conditions and under conditions of flow.

### 2.3. Assessment of the Hemocompatibility of the Adsorber Particle

Hemocompatibility of adsorber particles is crucial for potential future applications in the clinical situation. The ISO 10993-4 norm recommends specific tests to examine the effect of extracorporeal adsorber products in contact with blood [27]. In accordance with these guidelines, we analyzed the effects of the absorber particle on (i) complement factor 5a (C5a) production as a read-out for complement cascade activation, (ii) thrombin-antithrombin III (TAT) complex levels as a read-out for coagulation activation, and (iii) the thrombocyte concentration as a read-out for thrombocyte activation and clotting. Furthermore, leukocyte counts (iv) were quantified as an additional hematology parameter. In comparison, two commercially available adsorber particles used in hemoperfusion and in their “non-primed” state, were checked for their hemocompatibility properties (adsorbers A and B).

**Figure 4:**
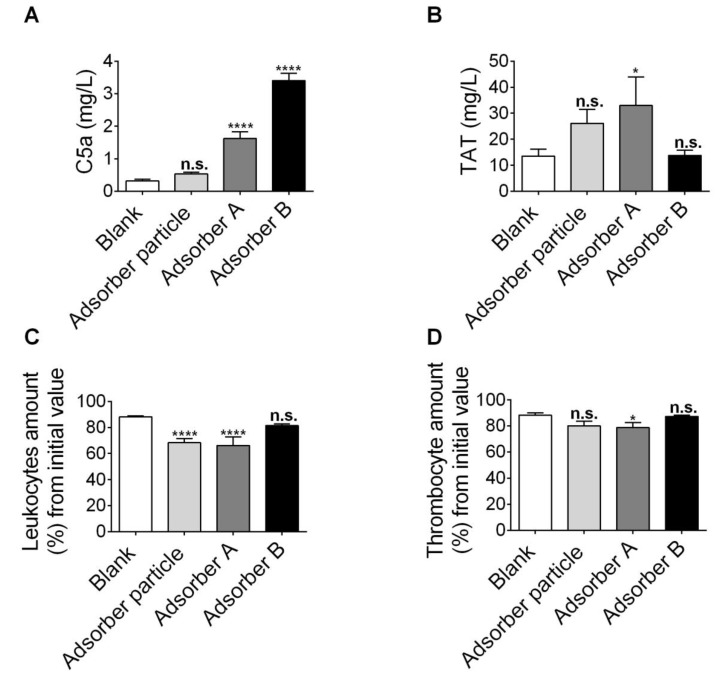
Hemocompatibility assessment of the newly developed adsorber particle. Hemocompatibility was assessed after flowing human blood for 180 min through a cartridge filled with the adsorber particles at a flow rate of 12.6 mL/min. One approach without particles was used as a blank. (**A**,**B**) Quantification of complement component 5a (C5a) (**A**) and the thrombin antithrombin-complex III (TAT) (**B**) in blood after incubation with newly developed whole-blood adsorber, adsorber A or adsorber B particles, or without particles (blank), as indicated. (**C**,**D**) Relative leukocyte count (**C**) and thrombocyte count (**D**) in blood after incubation with the newly developed whole-blood adsorber, non-primed adsorber A or adsorber B particles, or without particles (blank), as indicated, and displayed in % from the initial value in blood. (**A**–**D**) Data are given as mean values ± S.E.M; *n* = 15 for blank, *n* = 12 for the newly developed adsorber particle, *n* = 9 for adsorber A, and *n* = 5 for adsorber B. * *p* < 0.05, **** *p* < 0.0001, n.s. = not significant. One-way ANOVA with Dunnet’s multiple comparison test were all compared to the blank.

C5a is an essential pro-inflammatory mediator that is cleaved from its precursor upon blood damage [28]. Adsorbers A and B significantly increased the amount of C5a compared to the negative control (blank), as shown in Figure 4A, while the newly developed particle did not alter the C5a levels. In addition, the TAT complex was not significantly changed by the novel adsorber particle and the adsorber B particle (Figure 4B). In contrast, the adsorber A particle significantly increased TAT complex levels (Figure 4B). The number of leukocytes showed a significant decrease upon incubation with the absorber developed in the current study as well as upon incubation with adsorber A, but not with adsorber B (Figure 4C). Furthermore, a small but significant decrease in the thrombocyte concentration was observed for adsorber A particles, but not for the absorber developed in the current study nor for adsorber B (Figure 4D).

In summary, the newly developed particle showed a better hemocompatibility compared to the commercially available adsorber A and B particles in a native, non-primed condition, and mainly performs better in avoiding C5a production via complement cascade activation in contact with blood.

## 3. Discussion

Presently, the quality of dialysis as extracorporeal therapy of CKD patients is dependent on the efficiency of diffusion and filtration of uremic toxins out of the blood through a polymer membrane in a dialysis solution [29]. In general, hemodialysis assists patients in compensating their impaired kidney function by removing uremic toxins from the blood. However, the current dialysis principle preferentially eliminates low molecular weight, hydrophilic substances from the blood, whereas the removal of hydrophobic uremic toxins in particular remains a challenge. These hydrophobic uremic toxins bind to plasma proteins, e.g., serum albumin, resulting in protein-bound uremic toxins with an overall molecular weight greater than the cut-off for conventional dialysis membranes [30]. As a consequence, hydrophobic uremic toxins, such as phenylacetic acid, p-cresyl sulfate, and indoxyl sulfate, accumulate in the blood of CKD patients over time, even despite regular hemodialysis treatments [2]. This accumulation of uremic toxins in the blood adversely affects the cardiovascular system [11,12,13], with cardiovascular disease the major cause of premature death of CKD patients [31]. 

Patients with liver failure have comparable problems to patients with CKD. Toxins, such as ammonia, false neurotransmitters, phenols, aromatic amino acids, and other substances, accumulate in the blood of these patients and early liver-support systems have focused on blood detoxification to reduce patient morbidity. Many therapeutic processes to enhance the removal of toxins have been tested in the past, e.g., hemodialysis, hemofiltration, exchange transfusion, and extracorporeal blood perfusion over activated charcoal, alone or in combination [32,33,34,35]. However, the majority of these techniques predominantly remove water-soluble substances, whereby protein-bound substances accumulate in liver failure [36]. Patients suffering from acute deterioration of chronic liver disease have a mortality rate of above 60% without liver transplantation [37]. The fractionated plasma separation and adsorption (FPAD) system Prometheus^TM^ is a common extracorporeal system for the treatment of acute liver failure that significantly normalizes the serum levels of conjugated bilirubin, bile acids, ammonia, cholinesterase, creatinine, urea, and blood pH [21]. The Prometheus^TM^ system combines fractionated plasma separation and adsorption (FPAD) with a high-flux hemodialysis in an extracorporeal detoxification system [21,38]. Initial investigations of our group, in which CKD patients on dialysis (CKD-5D) were treated using the FPAD technique for 5 h, showed increased hydrophobic uremic toxin removal rates compared with conventional high-flux hemodialysis, with the removal rates increased by 130% for phenylacetic acid, 127% for p-cresyl sulfate, and 187% for indoxyl sulfate. There was no observed effect on safety parameters like cortisol, triiodothyronine, or total plasma protein [16]. However, the plasma separation approach causes high costs and is therefore not suitable for regular extracorporeal therapy of end-stage renal disease (ESRD) patients.

Furthermore, specific types and shapes of zeolite adsorbers have been examined for creatinine binding capacity [39], and others have shown that hexadecyl-immobilized cellulose beads can efficiently adsorb protein-bound uremic toxins [40]. Also, AST-120 is an orally administered, intestinal, activated carbon adsorbent particle that adsorbs uremic toxins like indole as a precursor of indoxyl sulfate. AST-120 was shown to improve the uremic condition in CKD patients and prolong the time to initiation of hemodialysis. More specifically, a decrease in serum indoxyl sulfate was observed in a dose-dependent manner by reducing the absorption of indole from the gastrointestinal tract in the presence of AST-120 [41,42]. However, creating whole-blood adsorbers for the chronic therapy of CKD patients still poses a challenge. Recently, Pavlenko et al. showed the efficient removal of uremic toxins from human plasma after 4 h incubation with a carbon-based adsorbent in static conditions [43]. In this study, we developed a highly porous, whole-blood-hemocompatible microparticle with an efficient adsorption capacity towards hydrophobic uremic toxins under flow conditions for the treatment of patients with CKD. The newly developed bifunctional whole-blood adsorber particle is specifically designed for the removal of hydrophobic, protein-bound uremic toxins and consists of a hydrophobic, porous, activated charcoal core and a hydrophilic coating with PVP. Due to the hydrophobic, porous core, hydrophobic uremic toxins may diffuse into the activated charcoal and bind there. PVP was chosen as the hydrophilic coating to counteract the binding of plasma proteins to the particle [44]. This complements the size-mediated exclusion of plasma proteins from the hydrophobic core by the pore size of the particle. Additionally, the coating with PVP was selected to induce hemocompatibility of the particles for whole-blood applications, as interference with protein adsorption also prevents downstream biological reactions [45].

Activated charcoal has already been used for the adsorption of methionine, tyrosine, and phenylalanine, as well as arsenic, selenium, and mercury [24,46]. Also, it is a well-established treatment option for gastrointestinal decontamination upon acute overdose [47] due to its hydrophobicity and large surface area and porosity, which favour the high adsorptive capacity. So far, however, studies have indicated that hemoperfusion over uncoated, activated charcoal was unacceptable because of excessive blood damage [48]. To avoid blood damage, we have previously screened different coatings for the activated charcoal (data not shown). From that screening, we selected PVP for the coating in this study, since previous studies have already shown an excellent biocompatibility of dialysis membranes blended with PVP [49,50,51]. Also, PVP in combination with iodine was already shown to demonstrate an acceptable tissue tolerability [52]. Further, PVP was tested earlier as a surface coating for silver nanoparticles and showed good hemocompatibility with erythrocytes [25]. Also, coating of hydrogel with PVP resulted in the decreased activation of platelets during contact with blood under flow, and its hemocompatibility was also confirmed in a small animal model [53]. Therefore, in our study, PVP was tested as particle coating with the aim to improve the hemocompatibility of activated charcoal, while simultaneously retaining the particle’s high adsorption capacity towards hydrophobic compounds. Therefore, different ratios of monomer (PVP) and cross-linker (ethylene glycol dimethacrylate) were tested for the charcoal coating as a first step to retain a particle with a high adsorption capacity (data not shown). 

The optimized whole-blood adsorber particle showed a very strong adsorption capacity for the analyzed hydrophobic uremic toxins phenylacetic acid, p-cresyl sulfate, and indoxyl sulfate, with a 92–97% adsorption of these toxins from blood after 30 min of incubation. In the recirculation experiment, 60 and 240 min of flow resulted in an adsorption of 84–96% and 95–100%, respectively. This revealed a high adsorber capacity of the developed whole-blood adsorber particle towards phenylacetic acid, p-cresyl sulfate, and indoxyl sulfate, also in conditions of flow, with a flow rate and incubation time comparable to the clinical dialysis situation. Our adsorber particle thereby exceeded the performance of hexadecyl-immobilized cellulose beads recently shown to adsorb indoxyl sulfate in vitro for 55.9 ± 1.4% [40]. Also, the adsorption performance of our particles exceeded that of CMK-3 and the commercially available Norit A Supra, both carbon-based sorbents recently shown to adsorb indoxyl sulfate to ≈80% and ≈90% in vitro, respectively [43]. Furthermore, our particles were at least comparable in adsorption performance to a hemocompatible, an activated carbon monolith recently demonstrated by Sandeman et al. as highly promising in binding p-cresyl sulfate and indoxyl sulfate from human plasma and whole blood [54]. In addition, the removal of phenylacetic acid, p-cresyl sulfate, and indoxyl sulfate from blood was more efficient by our particles compared to the commercially available FPAD system Prometheus^TM^, as well as compared to conventional high-flux hemodialysis, which could reduce the plasma concentrations of these uremic toxins in CKD patients by factors of 6.2, 3.4, and 4.6 (FPAD), respectively, and by factors of 2.7, 1.5, and 1.6 (conventional high-flux hemodialysis), respectively [16]. All combined, our adsorber particle showed a high adsorption capacity towards protein-bound uremic toxins, exceeding that of currently available adsorber systems, as well as conventional hemodialysis techniques. The uremic toxins phenylacetic acid, p-cresyl sulfate, and indoxyl sulfate are important representatives of the group of hydrophobic, protein-bound uremic toxins since they highly accumulate in CKD patients, even despite regular dialysis [2], and have been associated with increased cardiovascular risk in these patients [11,12,13]. Thus, our results indicate that the bifunctional, whole-blood adsorber particles may be very useful in the removal of hydrophobic uremic toxins from the plasma of CKD patients with the ultimate goal to reduce mortality in CKD patients. In contrast to the efficient binding of hydrophobic uremic toxins, the particles did not significantly adsorb serum albumin, which is an important finding, as albumin adsorption may reduce the adsorption capacity of the particles towards uremic toxins over time by narrowing the particle pores. 

Hemocompatibility in contact with the patient’s blood is still a major challenge during the development of new methods for the treatment of CKD patients. The hemocompatibility of the newly developed particles was thus tested according to the ISO norm 10993-4. This ISO norm recommends specific assays to evaluate potential adverse effects of biomaterials in contact with blood, specifically also for extracorporeal adsorber products [27]. As such, the ISO norm 10993-4 advises the inspection of effects of extracorporeal adsorber particles on in vitro thrombosis, and this is done by studying (i) coagulation (e.g., by TAT quantification); (ii) thrombocyte activation (e.g., by quantifying thrombocyte counts); and (iii) complement cascade activation, with C5a being one of the most important complement-derived pro-inflammatory mediators [55,56,57]. Increased TAT amounts are associated with a hypercoagulable state, with patients predisposed to thrombotic events showing elevated concentrations of TAT [58]. Further, thrombocytes are essential for blood clotting, and as blood clots reduce the amount of circulating thrombocytes, the thrombocyte count is recommended as a second parameter to provide information on pro-thrombotic effects [27]. Finally, leukocyte counts are suggested as an additional hematological control parameter, although according to the ISO 10993-4 norm, not absolutely required for evaluating the hemocompatibility of adsorber particles in blood contact [27]. Based on these ISO recommendations and the fact that contact between blood and particular biomaterials has previously been shown to trigger a complex series of events including TAT elevation, C5a production, and coagulation activation/inactivation [57,59], we focussed on these parameters in our hemocompatibility study to evaluate the potential of the newly developed adsorber particle for future clinical applications.

In this study, the marker for activation of the coagulation system and complement activation showed no significant difference after incubating blood with the newly developed particles under flow condition when compared to the control, whereas at least one of these parameters was significantly increased by the commercially available adsorber particles A and B. Furthermore, although the leukocyte concentration was significantly decreased by the newly developed adsorber particles to a comparable extent, as observed for commercial adsorber A particles, thrombocyte concentrations were not significantly decreased by the new adsorber particle in comparison to the control condition. Combined, these data suggest a good hemocompatibility of the newly developed whole blood adsorber particles, also in comparison with the commercially available adsorber particles in their non-primed state. The latter adsorbers have already been tested in the clinic for the treatment of patients with acute intoxication, sepsis or for patients with liver failure, and hypercholesterinemia [21,22]. Of note, for the assessment of hemocompatibility, identical conditions for all adsorber particles were selected, discarding any potentially required pre-treatment of the adsorbers. As such, the adsorber A was not primed with a recommended special buffer, as described in the according instructions for use (IFU), which may reduce hemocompatibility properties compared to primed adsorber A as used in clinical settings. This also suggests that further testing using differential priming conditions for our adsorber particles might even further improve their hemocompatibility properties.

All combined, the properties of the newly developed adsorber particle in comparison with those of commercial adsorber particles predestine our adsorber for clinical applications regarding the removal of hydrophobic uremic toxins from the blood of CKD patients, or by extension, from patients suffering from acute intoxication, sepsis, liver failure, or hypercholesterinemia. Whether the developed particles may trigger eosinophil increases due to allergic responses remains to be investigated in future in vivo studies, although PVP, used as a coating on our particles, has been reported to be non-allergenic, even after long-term exposure [60]. Furthermore, since certain drugs may also be removed through particle binding depending on the properties of the drug (e.g., hydrophobicity and size), the effect of adsorber particles on daily drug dosing in CKD patients remains to be investigated in more detail, and is an issue that has also been previously raised for alternative hemodialysis techniques [61]. Though outside the scope of this study, additional in vitro investigations of drug binding to our particle combined with in vivo pharmacokinetic data of selected drugs will further shed light on the issue of drug dosing when using adsorber particles in combination with hemodialysis.

Since the developed adsorber particle is hemocompatible, it could be integrated into the existing dialysis machines using an additional cartridge in row. Combined with the fact that activated charcoal and PVP are both low-cost materials, which was an essential aspect in the development of the adsorber material, this enables the extra production costs to be kept as low as possible. Nonetheless, an increase in therapy costs cannot be avoided when combining conventional dialysis with adsorption, and a preferential treatment of selected groups of highest-risk patients, patients with highest uremic toxin concentrations, and those with cardiovascular complications could be considered to keep additional healthcare costs under control. Furthermore, compared with the available Prometheus^TM^ system, which requires an expensive plasma separation and a filtration approach before adsorption [21], our adsorber particle is hemocompatible with whole blood. This eliminates the need for plasma separation, and considerably reduces therapy costs compared with the Prometheus^TM^ system.

In summary, we have developed a bifunctional, hemocompatible-coated whole-blood adsorber particle that is highly efficient in adsorbing hydrophobic, protein-bound uremic toxins as increased in the blood of patients with CKD stage 5. The hydrophilic surface modification with PVP prevented adverse interactions of corpuscular blood components with the adsorber, but still allowed for efficient binding of the porous, activated charcoal core pores with hydrophobic solutes in the blood. This approach resulted in a hemocompatible adsorber with a very high adsorption capacity for hydrophobic uremic toxins. Therefore, the whole blood adsorber can be used for the separation of protein-bound uremic toxins in combination with dialysis. As these protein-bound uremic toxins highly accumulate in CKD patients, even despite regular dialysis [2], and as these toxins are associated with increased cardiovascular risk in these patients [11,12,13], the newly developed adsorber particles show great promise toward increasing the efficiency of current conventional dialysis techniques in clearing pathophysiological, hydrophobic uremic toxins from the blood and thereby reducing morbidity and mortality in CKD patients. In future, this bifunctional, whole blood adsorber particle has to be tested on adsorption performance, as well as potential side effects, as allergic reactions in vivo to confirm the in vitro results for its clinical translation. 

## 4. Conclusions

The newly developed whole blood adsorber particle is hemocompatible and showed a very high capacity of adsorbing hydrophobic uremic toxins due to a combination of (i) size exclusion, (ii) hydrophobicity, and (iii) hemocompatibility. Thus, the adsorber particle shows great promise toward increasing the efficiency of conventional dialysis techniques in clearing pathophysiological, hydrophobic uremic toxins from the blood and thereby reducing morbidity and mortality in CKD patients. Future investigations will focus on the verification of these results in in vivo conditions.

## 5. Materials and Methods

### 5.1. Synthesis of the Particles

Activated charcoal adsorbent particles (diameter 510 µm; pore size volume 0.7 cm^3^ g^−1^) were purchased from the supplier IBU-tec advanced materials AG (Weimar, Germany) for coating with the hydrophilic polyvinylpyrrolidone (PVP). By varying the ratios of the 1-vinyl-2-pyrrolidone (PVP)-monomer versus ethylene glycol dimethacrylate as a crosslinker, a total of 13 particle types were generated. For the final adsorber particle, 200 g of particles were suspended under constant stirring in 4 L of an aqueous solution containing 2 wt% PVP (Sigma-Aldrich, Taufkirchen, Germany) as a stabilizer in a 20 L glass reactor (BüchiGlasUster, Uster, Switzerland) to coat these activated charcoal cores. The solution was heated up to 40 °C, after which, 2.8 g of azo-bis-isobutyronitrile (Sigma-Aldrich, Taufkirchen, Germany) as an initiator; 720 g 1-vinyl-2-pyrrolidone (Sigma-Aldrich, Taufkirchen, Germany) as a monomer; and 288 g of ethylene glycol dimethacrylate (Sigma-Aldrich, Taufkirchen, Germany) as a crosslinker were added. Subsequently, the solution was stirred for 2.5 h and, after raising the temperature to 65 °C, stirred for 11 hours overnight. Next, the resulting particles were sedimented, washed with deionized water, and stored in an aqueous 2% PVP-solution at 4 °C.

### 5.2. Microscopic Particle Characterization

The cross sections of the charcoal adsorbent particles and the final coated particle were analyzed using scanning electron microscopy (SEM; Zeiss GeminSEM, Oberkochem, Germany). Particles were broken by using liquid nitrogen and afterwards transferred onto conductive tabs (Plano GmbH, Wetzlar, Germany) for imaging. The images were taken using an accumulation grid of 8 mm with an accelerating voltage of 5 kV at 20,000× magnification and a resolution of 1024 × 1024 pixels. 

### 5.3. Statement of Ethical Principles

Blood was drawn from healthy volunteers after full informed consent. All experiments conform to the principles outlined in the declaration of Helsinki [62]. The study was approved by the ethic committee from the RWTH Aachen University (EK 153/18, 06/2018). 

### 5.4. Quantification of Adsorbed Hydrophobic Uremic Toxins by Analytical Reversed Phase Chromatography

In a first set-up, the adsorption of uremic toxins by adsorber particles was examined using static incubation conditions. Here, 100 mg of adsorber particles were resuspended in 10 mL of human blood or water containing 0.5% bovine serum albumin (BSA; Sigma-Aldrich, Taufkirchen, Germany). Correspondingly, a control sample without adsorber particles was treated in the same manner as a reference for calculating the amount of adsorbed uremic toxins. A mixture of uremic toxins according to the concentration of the uremic toxins in patients with CKD stage 5 (phenylacetic acid (474.0 µg/mL) [4], p-cresyl sulfate (41.0 µg/mL) [63], and indoxyl sulfate (44.0 µg/mL) [64]) was added to the blood or the BSA solution and incubated at 37 °C for 15, 30, 60, 120, or 180 min, as indicated. Next, the mixture was centrifuged at 3000 rpm for 10 min at 4 °C to deposit the adsorber particles and the supernatant was transferred to cryotubes followed by the addition of 25 mg/mL of resorcin (Sigma-Aldrich, Taufkirchen, Germany) as an internal standard for subsequent chromatographic quantification purposes. The samples were stored at −80 °C until analysis. For the quantification of serum albumin binding to the adsorber particles, 100 mg of adsorber particles were resuspended in 10 mL water containing 60 g/L BSA (Sigma-Aldrich, Taufkirchen, Germany). After the indicated defined time points, 20 µL samples were collected and used for quantifying the protein concentration using the BioRad DC protein kit (Bio-Rad Laboratories GmbH, Munich, Germany). The data were normalized to a control sample without adsorber particles.

The resulting uremic toxin concentrations of the samples were quantified using analytical reversed-phase high-performance liquid chromatography (RP-HPLC) with a C18 column (Chromolith Performance^TM^ column 100 mm × 4.6 mm; Merck, Darmstadt, Germany). The mobile phase consisted of 4 mM tetra-n-butyl ammonium hydrogen sulfate (TBA) in a 1 M K_2_HPO_4_ buffer (pH 6.5) as solvent A, and ethanol (100%; pH 6.5) was used as solvent B. A total of 10 µL of sample was mixed with 200 µL of solvent A and was injected into the column using the Dionex HPLC system (Thermo Fischer Scientific, Darmstadt, Germany). For elution, a linear gradient of 8 to 40% solvent B was applied for 8 min with a flow rate of 2 mL/min. Uremic toxins were analyzed by recording the UV absorbance at λ_220_ by using the Dionex Chromeleon software (version 6.6; Thermo Fischer Scientific, Darmstadt, Germany). Extinction coefficients were derived from standard plots generated by loading known concentrations of synthetic phenylacetic acid, p-cresyl sulfate, and indoxyl sulfate eluating on the HPLC column. Using these extinction coefficients, the total amount of uremic toxins of the samples was quantified, being the fraction not bound to the adsorber particles. Then, based on the difference between the initial concentration and the non-bound fraction, the fraction that was adsorbed to the adsorber particles was calculated. 

In a second approach, the adsorption of uremic toxins by adsorber particles was examined in conditions of flow. Twelve grams of adsorber particles was packed into an XK column 16/20 (GE Healthcare, Munich, Germany) and equilibrated with water. Later, one litre of PBS solution containing a mixture of uremic toxins (phenylacetic acid (474.0 µg/mL) [4], p-cresyl sulfate (41.0 µg/mL) [63], and indoxyl sulfate (44.0 µg/mL) [64]) was pumped at a flow rate of 8 mL/min through the cartridge. After the indicated defined time points, 2 mL samples were collected at the outlet of the cartridge. The samples were stored at −80 °C until analysis. For each sample, 150 µL was mixed with 15 µL internal standard (containing 10 mg/L indoxyl-3a,4,6,7,7a-^13^C_6_ sulfate, 10 mg/L phenylacetic acid-1-^13^C, and 10 mg/L p-tolyl sulfate-d_7_) (Sigma-Aldrich, Germany). The amount of adsorbed uremic toxins was analyzed using an Aquity-UHPLC (C18 column, Waters, Eschborn, Germany) system coupled to a TQD mass spectrometer (Waters, Eschborn, Germany) with an electrospray ionization (ESI) interface. The mobile phase consisted of 0.1% formic acid in water as solvent A and 0.1% formic acid in acetonitrile was used as solvent B. A total of 10 µL of sample (including internal standard) was injected onto the column. For elution, a linear gradient of 10 to 90% solvent B was applied for 3 min with a flow rate of 0.45 mL/min. All acquired mass spectrometry raw data was pre-processed using the software QuanLynx 4.1 SCN627 (Waters, Eschborn, Germany).

### 5.5. Assessment of Hemocompatibility

Five grams of the optimized adsorber particles were packed in a polyethylene cartridge (self-made) with aqueous 0.9% NaCl solution. For analysis of the hemocompatibility, two commercially available adsorber particles from two different suppliers that are used for hemoperfusion were selected to compare their hemocompatibility with that of our new particle. One type of these commercially available reference particles was DALI, purchased from Fresenius Medical Care (DALI cartridge Type adsorbent kit 1500, Bad Homburg, Germany) (“adsorber A”). These particles were developed and accredited for low-density lipoprotein (LDL)-apheresis technology for the selective removal of atherogenic LDL and lipoprotein(a) from whole blood. The second particle type that we selected for comparison of hemocompatibility was Gambro Prismafex Adsorba™ C300 (adsorber B, purchased from Baxter-Gambro Dialysatoren GmbH (Hechingen, Germany). Of note, for the assessment of the hemocompatibility, identical conditions for all adsorber particles were selected for optimal comparison of the “non-primed” adsorber properties, not taking into account potential special pretreatment (“priming”) of the commercial adsorber particles, e.g., as prescribed for the DALI (adsorber A) particles according to the instructions for use (IFU).

The cartridges containing the particles were connected by a tubing system with a reservoir filled with 125.0 mL of human, heparin-anticoagulated healthy donor blood, the latter gently stirred for the entire time to prevent clotting. The cartridge containing adsorber A was equilibrated with aqueous 0.9% NaCl solution; the cartridges containing particles developed and optimized in the current study or adsorber B particles were equilibrated with water. After flushing the cartridges and tubing of the system with 75 mL 0.9% NaCl, the blood was pumped through the cartridges using a flow rate of 12.6 mL/min for 180 min at 37 °C. Samples were collected before the beginning of the experiment (t = 0 min) and after 180 min (t = 180 min) at the outlet of the cartridge and stored at −80 °C until analysis. The samples from t = 0 min and t = 180 min were used to calculate the increase or decrease of the tested parameters below. Additionally, one approach without particles was used as blank. 

The system was continuously visually inspected for clotted blood during the testing procedure. Furthermore, the numbers of leukocytes and thrombocytes were quantified using the K-4500 from Sysmex (Norderstedt, Germany). Also, thrombin antithrombin complex III (TAT) and complement component 5a (C5a) of blood samples were quantified by using the Freedom EVOlyzer (Tecan, Männerdorf, Switzerland) and chemiluminescent enzyme-linked immunosorbent assays according to the manufacturers’ instructions (C5a ELISA Kit EIA-3327, DRG, Marburg; Germany; Enzygnost^®^ TAT micro, Siemens, Marburg; Germany).

### 5.6. Statistics

All data were statistically analyzed using commercially available software packages (SPSS 23, IBM; GraphPad Prism 6, GraphPad Software Inc.; La Jolla, CA, USA). Data were expressed as the mean value of a minimum of three independent experiments ± standard error of the mean. Results of repeated measurements were compared using a repeated measurements analysis of variance (one-way ANOVA). In the case of significant results, post-hoc testing was performed using the Dunnet-test for multiple measurements. * *p* < 0.05 was considered statistically significant.
